# Awareness of neurocysticercosis: A study from northwest India

**DOI:** 10.4103/0972-2327.78046

**Published:** 2011

**Authors:** Mohit Girotra, Chanchal Gera, Rtika Ryfka Abraham, Rajat Gauba, Tino Bansal, Parmdeep Kaur, Yashpal Singh, Jeyaraj D. Pandian

**Affiliations:** Department of Neurology, Christian Medical College, Ludhiana, Punjab, India; 1 Department of Medicine, Christian Medical College, Ludhiana, Punjab, India; 2 Departments of Social and Preventive Medicine and Betty Cowan Research and Innovation Centre, Christian Medical College, Ludhiana, Punjab, India

**Keywords:** Attitude, awareness, knowledge, neurocysticercosis

## Abstract

**Background:**

Neurocysticercosis (NCC) is a common cause of epilepsy in developing countries. In order to plan and implement prevention programs, it is essential to study the awareness of NCC.

**Objective:**

To study the awareness of NCC among patients with NCC and compare with age- and gender-matched controls without NCC.

**Setting and Design:**

Hospital based case–control study.

**Materials and Methods:**

Two hundred and fourteen subjects were studied (109 NCC patients, and 105 age- and gender-matched controls without NCC). The participants were selected from neurology and medical wards of a tertiary referral hospital in northwest India. They were interviewed by trained medical interns using a questionnaire.

**Results:**

64.2% of the NCC patients and 19% of control group had heard about NCC (*P* < 0.001). Knowledge regarding organ affected by NCC in the NCC group was 61.4% and in the control group was 80% (*P* = 0.09). Only 12.9% of the NCC group and none in the control group identified tape worm as a causative agent for NCC (*P* = 0.092). Negative effects of NCC on marriage and social life were more often cited by the NCC group but in the control group it was towards education (*P* = 0.004).

**Conclusions:**

The awareness of NCC was poor in both the groups. Educational programs are needed to improve the awareness about NCC among the patients and the public.

## Introduction

Neurocysticercosis (NCC) is a major public health problem in India, Latin America and southeast Asian countries.[1] NCC has become an important emerging infection in the industrialized world due to increased travel and immigration of people from endemic areas.[2]

About 40 million of the 50 million people with epilepsy in the world reside in the developing world where epilepsy continues to be a highly stigmatizing condition.[3–6] In most developing countries, 10% of acute neurological cases are patients with NCC.[7]

Epilepsy and raised intracranial hypertension are the most common clinical forms of NCC.[7,8] It accounts for 50% patients presenting with partial seizures in some parts of India.[3,9] Single enhancing CT lesions (SECTL) are the commonest radiological abnormality in patients with new onset partial seizures in the endemic regions in India.[9] Epilepsy caused by NCC consequently represents an enormous expenditure for the developing world in terms of human suffering, lost production, and cost of anticonvulsants and the utilization of medical resources.[10]

The control measures recommended to combat this disease include improvements in hygiene, sanitation, control of pig slaughter, mass tenicidal chemotherapy, and health education.[11] To develop effective educational interventional programs for NCC, it is essential to know the level of awareness about the disease. In India, there is no paucity of literature on clinical manifestations, diagnosis and prognosis of NCC in adults and children. However, there is only limited information available in regards to awareness of NCC from the Indian subcontinent.

We aimed to study the awareness of NCC among patients, and compare with the subjects without NCC.

## Materials and Methods

This hospital based study was conducted at a tertiary referral hospital in northwest India.

All consecutive patients aged ≥16 years with NCC, who were admitted in the Department of Neurology between January 2005 and January 2006, were eligible for inclusion. Only patients with new onset NCC presenting with neurological symptoms for the first time were included. The diagnosis of NCC (definitive or probable NCC based on the absolute, major, minor and epidemiologic criteria) or SECTL (seizures without raised intracranial pressure or focal deficits, and absence of systemic illness; solitary enhancing lesion in the CT of ≤2 cm without midline shift) was made according to the published criteria.[12,13]

### Questionnaire

The questionnaire was adapted from previous studies, which was modified by authors to suit the local socioeconomic practices.[7,11] The first section of the questionnaire gathered demographic information about the patient along with their religion, education and income. The second section of the questionnaire tested the subjects’ knowledge about the disease – its symptoms, diagnosis and treatment options (western medicine, Indian medicine, antiepileptic drug, cysticidal drug, etc.). Also, in this section, the subjects were interviewed on issues like effect of their disease on education, marriage and social behavior. We also collected information about the actual symptomatology they encountered in their illness and also noted down their computed tomography/magnetic resonance imaging (CT/MRI) findings along with the type of treatment they received.

The survey instrument was pre-tested using a sample of 20 subjects (10 cases and 10 controls). A few necessary changes were made in the questionnaire based on the pre-testing.

Trained medical interns interviewed the subjects using a structured, pre-tested open-ended questionnaire. Questions were asked during a one to one interview in local vernacular language (i.e. Punjabi or Hindi), and the interviewer intervened only to clarify a question, when needed. Effort was made to interview the NCC patients before the diagnosis was discussed with them.

### Control subjects

The controls were consecutive patients admitted to medical/neurology wards without CT/MRI lesions consistent with those of NCC. These patients underwent imaging for other neurological symptoms (e.g. stroke, headache, vertigo, etc.). The same questionnaire was used to interview the control subjects.

Statistical analyses were done using SPSS version 16. Fisher’s exact test and Chi-square test were used to compare the different variables between the NCC and the control group. The means of the continuous variables were compared between the two groups using *t*-test. *P* < 0.05 was considered significant.

## Results

A total of 214 individuals (109 cases and 105 controls) were interviewed using the questionnaire.

### Demographic information

The two groups were matched for age and gender (age: NCC group, 28.3 ± 12.9 years; control group, 29.8 ± 12.8 years; *P* = 0.50; gender: NCC group, men 63; control group, men 58; *P* = 0.40). Majority of the respondents in both NCC (84, 77%) and the control groups (93, 88.5%) were from urban areas. The NCC patients were more educated (83.4%) as compared to those of the control group (64.7%).

### Knowledge of neurocysticercosis

Seventy (64.2%) NCC patients and 20 (19%) in the control group had heard about NCC (*P* < 0.001) [Table 1]. The rest of the analysis regarding knowledge, attitude and practice was confined to the above group of patients and controls. Knowledge regarding organ affected in NCC was 61.4% and 80% (*P* = 0.09) in the NCC and control groups, respectively [Table 1]. Only 12.9% in the NCC group and none in the control group identified tape worm as a causative agent for NCC (*P* = 0.092) [Table 1]. In the NCC group, 35.7% of participants believed that NCC spreads by eating pork and 30% in the control group believed that it spreads by drinking contaminated water [Table 1]. The NCC group subjects were more knowledgeable regarding the symptoms of NCC than the control subjects [Table 1].

**Table 1:** Comparison of knowledge about causative agent in neurocysticercosis and control groups

	NCC (N = 70) Yes n (%)	Control (N = 20) Yes n (%)	*P*
What do you know about NCC?			
It affects brain	43 (61.4)	16 (80)	0.099
It affects brain and other organs	16 (22.9)	1 (5.0)	0.062
It is due to some worm which affects brain	11 (15.7)	0 (0)	0.052
Which agent is responsible for this disease?			
Worm	38 (54.3)	6 (30.0)	0.055
Bacteria	3 (4.3)	0 (0)	0.466
Don’t know	31 (44.3)	14 (70)	0.043
Do you know the name of worm?			
Tape worm	9 (12.9)	0 (0)	
Round worm	61 (87.1)	20 (100)	0.092
How does it spread?			
Food	17 (24.3)	11 (55)	0.009
Fresh vegetables	15 (21.4)	6 (30)	0.424
Contaminated water	21 (30)	6 (30)	1.00
Eating pork	25 (35.7)	1 (5)	0.005
Others	2 (2.9)	0 (0)	0.603
Don’t know	17 (24.3)	1 (5)	0.048
What are the symptoms of NCC?			
Headache	39 (55.7)	8 (40)	0.215
Seizures	59 (84.3)	10 (50)	0.001
Paralysis	6 (8.6)	3 (15)	0.318
Unconsciousness	22 (31.4)	1 (5)	0.012
Don’t know	8 (11.4)	6 (30)	0.043

NCC, Neurocysticercosis

### Attitude toward neurocysticercosis

Seventy-four percent in the NCC group and 45% in the control group mentioned that they would allow their child to play with NCC patients (*P* = 0.013) [Table 2]. Thirty percent of the respondents in the control group thought that NCC was contagious as compared to 8.6% in the NCC group (*P* = 0.013) [Table 2]. Positive attitude toward employment and negative attitudes toward marriage and social life were commonly seen in the NCC group [Table 2]. However, in the control group, 90% had negative attitude toward education (*P* = 0.004) [Table 2].

**Table 2:** Responses of attitude toward neurocysticercosis in both the groups

	NCC (N = 70) Yes n (%)	Control (N = 20) Yes n (%)	*P*
Will you allow your child to play with a boy/girl with NCC?	52 (74.3)	9 (45)	0.013
Is this disease contagious?	6 (8.6)	6 (30)	0.013
Will you employ a person with NCC?	35 (50)	10 (50)	1.00
Does NCC affect education?	39 (55.7)	18 (90)	0.004
Does NCC affect marriage?	41 (58.6)	8 (40)	0.141
Does NCC affect social life?	44 (62.9)	6 (30)	0.009

NCC, Neurocysticercosis

### Treatment and prevention of neurocysticercosis

Majority of the NCC patients (77.1%) believed in allopathic treatment as compared to 35% in the control group (*P* < 0.001) [Table 3]. Knowledge regarding use of cysticidal drugs was also low in both the groups (NCC 8.6% and control 0%; *P* = 0.211) [Table 3]. A large proportion of NCC patients (45.7%) but none in the control group believed in ayurvedic treatment (*P* < 0.001) [Table 3]. Majority of the respondents in the control group (65%) were not aware of any treatment available for NCC (*P* < 0.001) [Table 3]. Regarding prevention of NCC, 65% in the control group believed that by drinking clean water they would prevent spread of NCC and 34.3% in the NCC group believed that NCC can be controlled by avoiding eating pork [Table 3].

**Table 3:** Responses regarding knowledge of treatment and prevention of neurocysticercosis (NCC) between the two groups

	NCC (N = 70) Yes n (%)	Control (N = 20) Yes n (%)	*P*
What are the treatments available?			
Homeopathic (Indian medicine)	4 (5.7)	0 (0)	0.359
Antiepileptic drugs	5 (7.2)	0 (0)	0.271
Allopathic (western medicine)	54 (77.1)	7 (35)	<0.001
Cysticidal drugs	6 (8.6)	0 (0)	0.211
Ayurvedic (Indian medicine)	32 (45.7)	0 (0)	<0.001
Don’t know	10 (14.3)	13 (65)	<0.001
How can you prevent NCC?			
Clean water	28 (40)	13 (65)	0.048
Safe sanitation	4 (5.7)	3 (15)	0.181
Avoid pork	24 (34.3)	1 (5)	0.007
Pig control	4 (5.7)	1 (5)	0.693
Slaughter house regulations	2 (2.9)	0 (0)	0.603
Any other	1 (1.4)	0 (0	0.778
Don’t know	30 (42.9)	5 (25)	0.149

NCC, Neurocysticercosis

## Discussion

We explored the awareness of NCC in this study. Majority in the NCC group had heard about the disease and the knowledge regarding organ affected in NCC was good in both the groups. But the knowledge regarding the responsible causative agent for the NCC was poor in both the groups. Awareness regarding presenting symptoms of NCC was greater in the patient group. The NCC patients had negative attitude toward marriage and social life, while the control group had the same toward education. The knowledge regarding prevention of NCC was poor in both the groups.

There are only two studies reported in the literature regarding knowledge, attitude and practice toward NCC. The first one was a population based study of a rural community in Mexico, evaluating the health education before and after educational intervention.[11] The authors interviewed 1931 persons in 386 households.[11] The second study explored the awareness of taeniasis and NCC in a very small sample of 40 teachers in Delhi, India.[14]

In this study, there were numerous gaps in knowledge about various aspects of NCC in both the groups. Sixty-four percent of the NCC patients had heard about NCC as compared to 19% of participants in the control group. In the study from Mexico[11] and Delhi,[14] only 10 and 26% of the respondents, respectively, had heard about NCC. The NCC patients had a better knowledge regarding the organ affected in NCC but both the groups did not correctly name the causative agent, tapeworm. In the Mexican study, none of them knew about the causative agent before the educational intervention, which increased the awareness to 9.6% after the intervention.[11] In the Indian study also, none of the teachers knew about tapeworm as the causative agent of NCC.[14]

The overall attitudes of the participants were negative toward marriage and social life in the NCC group and toward education in the control group. None of the previous studies have explored this aspect of the disease. The epilepsy and seizure that majority of these patients have could be the reason for the negative attitudes seen in our cohort.[5,6]

The NCC patients were better aware about the symptoms of NCC than the control group subjects. Regarding the preferred treatment, majority in the NCC group favored allopathic (western) treatment but many also believed in ayurvedic treatment. In India, it is very often seen that for any neurological ailment, patients also take other indigenous treatments in addition to allopathic treatment.[5,6,15]

This study has limitations. Firstly, it is a hospital based study which has its own selection bias unlike population based studies. The results could be generalized to an urban community in India. Secondly, biological markers such as presence of teniasis in stools or of serological tests for patients, controls and their respective family members were not done. Thirdly, we also did not explore another important practice followed in North India by the NCC patients, i.e. visiting quacks who specialize in removing worms from the brain via the nose using a mouth-held suction apparatus. Fourthly, we cannot exclude a selection bias in NCC patients even though every effort was made to interview before they were informed and educated about the disease. The NCC group was more educated than the control group. This would have influenced the responses in the NCC patients. Despite its limitations, this is the first Asian study to explore the awareness and attitudes toward NCC among the patients.

In conclusion, the awareness of NCC is poor in both the groups. Educational programs are needed to improve the awareness about NCC among the patients and the public.
